# Continuous Tracking of Task Parameters Tunes Reaching Control Online

**DOI:** 10.1523/ENEURO.0055-22.2022

**Published:** 2022-07-20

**Authors:** Antoine De Comite, Frédéric Crevecoeur, Philippe Lefèvre

**Affiliations:** Institute of Information and Communication Technologies, Electronics and Applied Mathematics (ICTEAM), Institute of Neuroscience (IoNS), UCLouvain, 1348 Louvain-la-Neuve, Belgium

**Keywords:** dynamical control, online feedback control strategy, reaching movement, target switching

## Abstract

A hallmark of human reaching movements is that they are appropriately tuned to the task goal and to the environmental context. This was demonstrated by the way humans flexibly respond to mechanical and visual perturbations that happen during movement. Furthermore, it was previously showed that the properties of goal-directed control can change within a movement, following abrupt changes in the goal structure. Such online adjustment was characterized by a modulation of feedback gains following switches in target shape. However, it remains unknown whether the underlying mechanism merely switches between prespecified policies, or whether it results from continuous and potentially dynamic adjustments. Here, we address this question by investigating participants’ feedback control strategies in presence of various changes in target width during reaching. More specifically, we studied whether the feedback responses to mechanical perturbations were sensitive to the rate of change in target width, which would be inconsistent with the hypothesis of a single, discrete switch. Based on movement kinematics and surface EMG data, we observed a modulation of feedback response clearly dependent on dynamical changes in target width. Together, our results demonstrate a continuous and online transformation of task-related parameters into suitable control policies.

## Significance Statement

Humans can adjust their control policy online in response to changes in the goal structure. However, it was unknown whether this adjustment resulted from a switch between two policies, or from dynamic and continuous adjustments. To address this question, we investigated whether online adjustments were tuned to dynamic changes in goal target which varied at different rates. Our results demonstrated that online adjustments were tuned to the rate of change in target width, suggesting that human reaching control policies are derived based on continuous monitoring of task-related parameters supporting online and dynamic adjustments.

## Introduction

Humans can execute reaching movements in various environments in the presence of unexpected disturbances such as visual or mechanical perturbations, that can interfere with their ability to succeed. Indeed, a large body of work characterized human control policies during reaching in presence of step mechanical (Knill et al., 2011; Nashed et al., 2012; Lowrey et al., 2017; Cross et al., 2019), visual (Georgopoulos et al., 1981; Soechting and Lacquaniti, 1983; Prablanc and Martin, 1992; Sarlegna and Mutha, 2015), or vestibular perturbations (Keyser et al., 2017; Oostwoud Wijdenes et al., 2019). Crucially, the perturbations used in these experiments recruited feedback circuits without altering the limb dynamics, which allowed establishing the dependency of the control policy on task requirements. These results highlighted that reaching control policies flexibly adapted to a wide variety of contexts while relying on different sensory modalities.

To capture this feature, the control of upper limb reaching movements can be modeled in the framework of optimal feedback control (OFC). This theory posits that reaching control policies optimize a performance index captured by a cost-function consisting of a weighted combination of motor cost and state-dependent movement penalties. This cost-function encompasses the task requirements by determining how to control the limb optimally with respect to this goal (Todorov and Jordan, 2002; Todorov, 2004). OFC has been used to model a diverse set of perturbation paradigms and established the flexibility of goal-directed feedback control in humans (Diedrichsen, 2007; Izawa and Shadmehr, 2008; Diedrichsen and Dowling, 2009; Omrani et al., 2013; Nashed et al., 2014; Scott, 2016).

It is important to realize that in most studies, the planning and control phases have been dissociated. Indeed, it was often assumed that the movement goal is selected before executing the corresponding control policy (Wong et al., 2015). In the OFC framework, the dissociation of planning and execution corresponds to the assumption that the feedback gains, and therefore the control policy, are derived before movement. In this view, it is unclear whether and based on which variables can the nervous system update control of an ongoing movement following changes in task-related parameters altering the movement goal, thereby implying a novel cost and requiring an adjustment of the policy. Crucially, we must distinguish perturbations as target jumps or mechanical loads, which computationally can be handled by altering the state vector without changing the controller, from changes in task requirements such as the structure of the target, that impose a change in the controller itself.

We recently demonstrated that the goal-directed policy used during reaching was adjusted online in response to changes in target width (De Comite et al., 2021). Here, we sought to investigate whether such adjustments reflected participants’ ability to switch between two prespecified control strategies, or whether they resulted from a feedback system considering continuous changes in the goal structure, and responded accordingly.

We addressed this question in two experiments where participants had to perform reaching movements toward a target the width of which could gradually decrease at different rates during movement, corresponding to a continuous modification of the target redundancy along its main axis. Two alternative hypotheses can be formulated: if adjustments in control policy do not integrate the dynamical changes in target, we expect to see stereotyped switches in behavior and feedback responses across conditions reflecting switches between two extreme cases (corresponding to maximal and minimal target widths). On the contrary, if dynamic changes are monitored, different rates of changes in target width should evoke different amounts of modulation in feedback responses. In agreement with the second alternative, we observed across the two experiments that participants adjusted their response to the rate of change in target width. Together, our results demonstrate the existence of a feedback mechanism conveying continuous information about task-parameters and adjusting the control policies dynamically.

## Materials and Methods

### Participants

A total of 24 right-handed participants were recruited for this study and were enrolled in one of the two experiments. Fourteen participants (10 females) ranging in age from 18 to 30 years old took part to experiment 1. The second group performed experiment 2 and included 10 right-handed participants (5 females) ranging in age from 19 to 27 years old. Participants were naive to the purpose of the study, had normal or corrected vision, and had no known neurologic disorder. The ethics committee of the local university approved the experimental procedures and participants provided their written informed consent before the experiment.

### Experimental paradigm

Participants were seated on an adjustable chair in front of a Kinarm end-point robotic device (KINARM) and grasped the handle of the right robotic arm with their right hand. The robotic arm allowed movements in the horizontal plane and direct vision of both the hand and the robotic arm was blocked. Participants sat such that, at rest, their arm was approximately vertical and their elbow formed an angle of ∼90°.Their forehead rested on a soft cushion attached to the frame of the robot. A semi-transparent mirror, located above the handle and reflecting a virtual reality display (VPixx, 120 Hz) allowed participants to interact with visual targets. A white dot of 0.5-cm radius aligned to the position of the right handle was displayed throughout the whole experiment.

### Experiment 1

**Figure 1.**
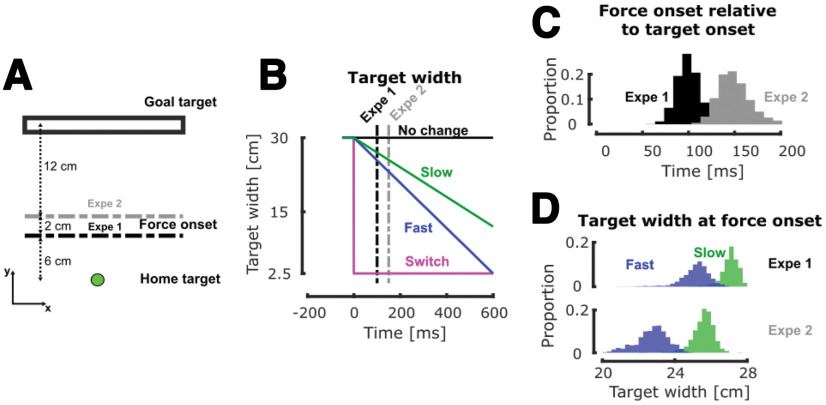
Experimental paradigms. ***A***, Schematic representation of the task paradigm. Participants had to perform reaching movement from the home target to the goal target, initially represented as a 30-cm-wide rectangle. During movement, they could experience mechanical step forces triggered in position at 6 cm (experiment 1, black line) or 8 cm (experiment 2, gray line) from the home target and visual changes in target width (triggered as they exited the home target). ***B***, Evolution of the target width with respect to time in the different target conditions. The time axis is aligned on the visual perturbation onset defined by the onset of movement. The vertical dashed lines represent the median force onsets for experiments 1 and 2. ***C***, Histograms of the distribution of the time interval between the visual and mechanical perturbation onset (respectively, target and force onset) across all participants and conditions in experiments 1 (black) and 2 (gray). ***D***, Histograms of the distribution of the target width at force onset for the two dynamical conditions (fast in blue and slow in green) across all participants in experiments 1 (top) and 2 (bottom).

In this experiment, participants (*N* = 14) were instructed to perform reaching movements to a visual target initially represented as a wide rectangle (30 × 2.5 cm) located 20 cm away from the home target in the *y*-direction. The home target was a circle of 1.5 cm in diameter. The main axis of the rectangle was aligned with the *x*-axis and was orthogonal to the straight-line path from the home target to the center of the goal target (see Fig. 1*A*). Participants first had to bring the hand-aligned cursor in the home target displayed as a red circle that turned green as they reached it. After a random delay (uniform, between 1 and 2 s), the goal target was projected as a gray rectangle and participants could begin their movement whenever they wanted. There was no constraint on the reaction time. The exit from the home target was used as an event to determine reach onset, and starting then participants had to complete their movement between 350 and 600 ms to successfully complete the trial. The trial was successfully completed if (1) they reached the goal target within the prescribed time window; and (2) they were able to stabilize the cursor in it for 500 ms. The goal target turned green at the end of successful trials and red otherwise. To motivate the participants, a score corresponding to their number of successful trials was projected next to the goal target.

During movements, two types of perturbations could occur. The first one was a mechanical load consisting of a lateral step force applied by the robot to participants’ hand (53.6% of trials). The magnitude of this force was ±9 N aligned with the *x*-axis, with a 10-ms linear build-up. This force was triggered when the hand-aligned cursor crossed a virtual line parallel to the *x*-axis and located at 6 cm from the center of the home target (see Fig. 1*A*, horizontal black dashed line). This step force was switched off at the end of the trial. The second type of perturbation was a visual change in target width starting when participants exited the home target (51.2% of trials). Hereafter, we refer to the visual perturbation as the target condition. Participants had no information about the target condition before movement initiation. This change could either be an instantaneous change from a wide rectangle to a narrow square (switch condition; Fig. 1*B*, magenta) or a continuous change in target width either at a speed of –30 cm/s (slow condition; Fig. 1*B*, green) or at a speed of −45.8 cm/s (fast condition; Fig. 1*B*, blue). The speed of the fast condition was selected such that the target width at the end of the movement was similar to the switch conditions for the slowest correct movements. This was done to assess whether participants could anticipate the final width of the target and select a corresponding controller, which would produce identical responses in the fast and switch conditions. The decrease in target width stopped as participants entered the goal target. Importantly, the location of the center of the goal target did not change across conditions and was always aligned with the home target. Unperturbed and perturbed trials were randomly interleaved such that participants could not predict the occurrence and the nature of disturbances. Participants were instructed to reach the target as it was actually displayed. They started with a 25-trials training block to become familiar with the task, the timing constraints, and the force intensity of perturbation loads. Crucially, this training block did not contain any visual perturbation. After completing this training block, participants performed six blocks of 82 trials. Each 82-trials block contained: 38 trials without mechanical perturbation (20 with no target change and 6 for each target condition) and 44 trials with mechanical perturbation (20 with no target change and 8 for each target condition, equally likely for rightward and leftward mechanical perturbations). Participants performed a total of 492 trials, including 24 of each combination of perturbed condition (direction of the mechanical perturbation and target condition), see Table 1. Participants were compensated for their participation.

**Table 1:** Trials distribution for each block of the two experiments

	No change	Slow	Fast	Switch
No	20	6	6	6
Left	10	4	4	4
Right	10	4	4	4

### Experiment 2

We designed a second experiment which was a variant of the first one to assess reproducibility of the results in a slightly different version of the protocol, and also to investigate possible influence of the delay between the visual and mechanical perturbations on the modulation of feedback responses. Experiment 2 was almost identical to experiment 1, except that the mechanical perturbation was triggered when the hand-aligned cursor crossed a virtual line parallel to the *x*-axis and located at 8 cm (instead of 6) from the center of the home target (see Fig. 1*A*, gray dashed line). The intensity of this mechanical load was reduced compared with the main experiment (7 vs 9 N) to keep a similar success rate. All the other experimental parameters (target conditions, number of trials, and time constraints) were identical to those of experiment 1, see Table 1.

Since both visual and mechanical perturbations were triggered based on position threshold (respectively, when participants exited the home target and when they crossed a virtual line located at 6 or 8 cm from the center of the home target), there was some variability in the time span between these two perturbation triggers. The variability in this time span is represented in Figure 1*C*, black and gray for experiments 1 and 2, respectively, and had a median value of 96 ± 6.41 ms for experiment 1 and 145 ± 21.73 ms for experiment 2. As the target width in the slow and fast conditions were continuously changing with time, some variability was also present in the target width at the mechanical perturbation onset. In the fast condition, we observed a median value of 25.6 ± 0.3 and 23.31 ± 0.14 cm, while in the slow condition, we observed a median value of 27.1 ± 0.15 and 26.3 ± 0.54 cm, respectively, for experiments 1 and 2, represented in Figure 1*D* in blue (fast) and green (slow).

### Data collection and analysis

Raw kinematics data were sampled at 1 kHz and low-pass filtered using a fourth order double-pass Butterworth filter with cutoff frequency of 20 Hz. Hand velocity, acceleration and jerk were computed from numerical differentiation of the position using a fourth order centered finite difference.

Surface EMG electrodes (Bagnoli surface EMG sensor, Delsys INC.) were used to record muscles activity during movements. We measured the pectoralis major (Pect. Maj.) and the posterior deltoid (Post. Delt.) based on previous studies (Crevecoeur et al., 2019, 2020b; De Comite et al., 2021) showing in the same configuration that these muscles were stretched by the application of lateral forces, and therefore strongly recruited for feedback responses. Before applying the electrodes, the skin of participants was cleaned and abraded with cotton wool and alcohol. Conduction gel was applied on the electrodes to improve the quality of the signals. The EMG data were sampled at a frequency of 1 kHz and amplified by a factor of 10,000. A reference electrode was attached to the right ankle of the participant. Raw EMG data from Pect. Maj. and Post. Delt. were bandpass filtered using a fourth order double-pass Butterworth filter (cut-offs: 20 and 250 Hz), rectified, aligned to force onset, and averaged across trials or time as specified in Results. EMG data were normalized for each participant to the average activity collected when they maintained postural control against a constant force of 9 N (rightward for Pect. Maj., leftward for Post. Delt.) This calibration procedure was applied after the second and the fourth blocks.

### Statistical analyses

Data processing and parameter extractions were performed using MATLAB 2019a. We fitted linear mixed models (Brown and Prescott, 2006) to infer the effect of target conditions on different kinematics parameters and on the EMG activities. These models were fitted using the *fitlme* function and the formula used was the following:

$$Parameter_{ij}=\beta_{0}+\beta_{1}\text{ × }Condition+\alpha_{i}+\epsilon_{ij}.$$

In this formula, the fixed predictors were the intercept ( $\beta_{0}$) and the target condition ( $\beta_{1}$) while participants were included as a random offset ( $\alpha_{i}$). The individual residual of trial *j* for participant *i*, captured by $\epsilon_{ij}$ followed a normal distribution. Each target condition was associated with an integer number such that they were ordered in decreasing order of constraints on the final target (no change<slow<fast<switch) and that positive/negative values for the regressor $\beta_{1}$ indicate a decrease/increase of the measured parameter with the task difficulty. For these linear mixed model analyses that we performed, we reported the mean estimate of $\beta_{1}$, its SD, the *t* statistics for this estimate and the corresponding *p* value.

The continuous predictor for the condition can be seen as a nonlinear transform of task difficulty and the parameter $\beta_{1}$ can be interpreted as a slope, meaning that the more difficult the task is (with narrower target), the larger the feedback response. However, this approach can be criticized as the condition may also be considered as a categorical predictor. To address this concern, we also ran a discrete version of the linear mixed models where the target condition was defined as categorical. This categorical model confirmed the conclusion of the continuous one in all the conditions (results not shown). *Post hoc* tests between pairs of target conditions were performed using similar linear mixed model applied on the two compared target conditions. For these *post hoc* tests, we reported the mean estimate of $\beta_{1}$, its SD, the *t* statistics for this estimate, the corresponding *p* value, and the effect size defined as the standardized mean difference between two groups of independent observations (Lakens, 2013).

In order to determine whether the timing of the mechanical perturbation relative to the onset of visual change could modulate the feedback responses, we compared the results of experiments 1 and 2 as follows. We normalized the EMG activity by the intensity of the mechanical perturbations (9 and 7 N for experiments 1 and 2, respectively), and binned them within trial in the long-latency (LL; 50–100 ms following perturbation onset) and early-voluntary (VOL; 100–180 ms following perturbation onset; Pruszynski et al., 2008; Pruszynski and Scott, 2012). We then ran the following linear mixed effect models for each of these binned response value:

$$Parameter_{ij}=\beta_{0}+\beta_{1}*targetcondition+\beta_{2}*experiment+\alpha_{i}+\epsilon_{ij}.$$

The fixed predictors are the intercept ( $\beta_{0}$), the target condition ( $\beta_{1}$) and the experiment ( $\beta_{2}$, a proxy for the onset of mechanical perturbation) while participants were included as random offset ( $\alpha_{i}$). The individual residual of trial *j* for participant *i*, captured by $\epsilon_{ij}$ followed a normal distribution. As above, we verified that continuous and categorical definitions of the target condition yielded similar results and reported the statistics corresponding to the continuous predictor. *Post hoc* tests between pairs of conditions were performed using linear mixed models applied on the two compared target conditions.

We also investigated a potential learning or habituation effect across fast and slow conditions as participants did not encounter those trials in the training phase. In order to investigate the lag between the first and last trials in the dynamical conditions (namely, the slow and fast conditions that were not met during the training phase), we used a cross-correlation analysis applied on resampled data. We generated 1000 bootstrap samples from the individual acceleration profiles. For each of these samples, we computed the mean acceleration traces for the first and last trials and computed the cross-correlation between these two mean traces. We then extracted the peak value of this cross-correlation, corresponding to the lag between the two signals. The bootstrap resampling allowed us to obtain a distribution for this lag such that we could perform statistical analyses on it. Wilcoxon signed rank test was used to assess whether differences in lag were statistically different or not from zero.

In all our analyses, significance was considered at the level of *p* = 0.05 although we decided to exactly report any p-value that was larger than *p* = 0.005 as previously proposed (Benjamin et al., 2018).

## Results

### Experiment 1

Participants were asked to perform reaching movements to a target that was initially a 30-cm-wide rectangle, in all cases. During movement and in a random subset of trials, the target could either instantaneously turn into a 2.5-cm-wide square target (switch condition) or gradually decrease in width either at a high (fast condition) or low (slow condition) speed. Additionally, unexpected mechanical perturbations were used during movements to evoke rapid motor responses and investigate their properties in relation with the change in target width

#### Kinematics

**Figure 2.**
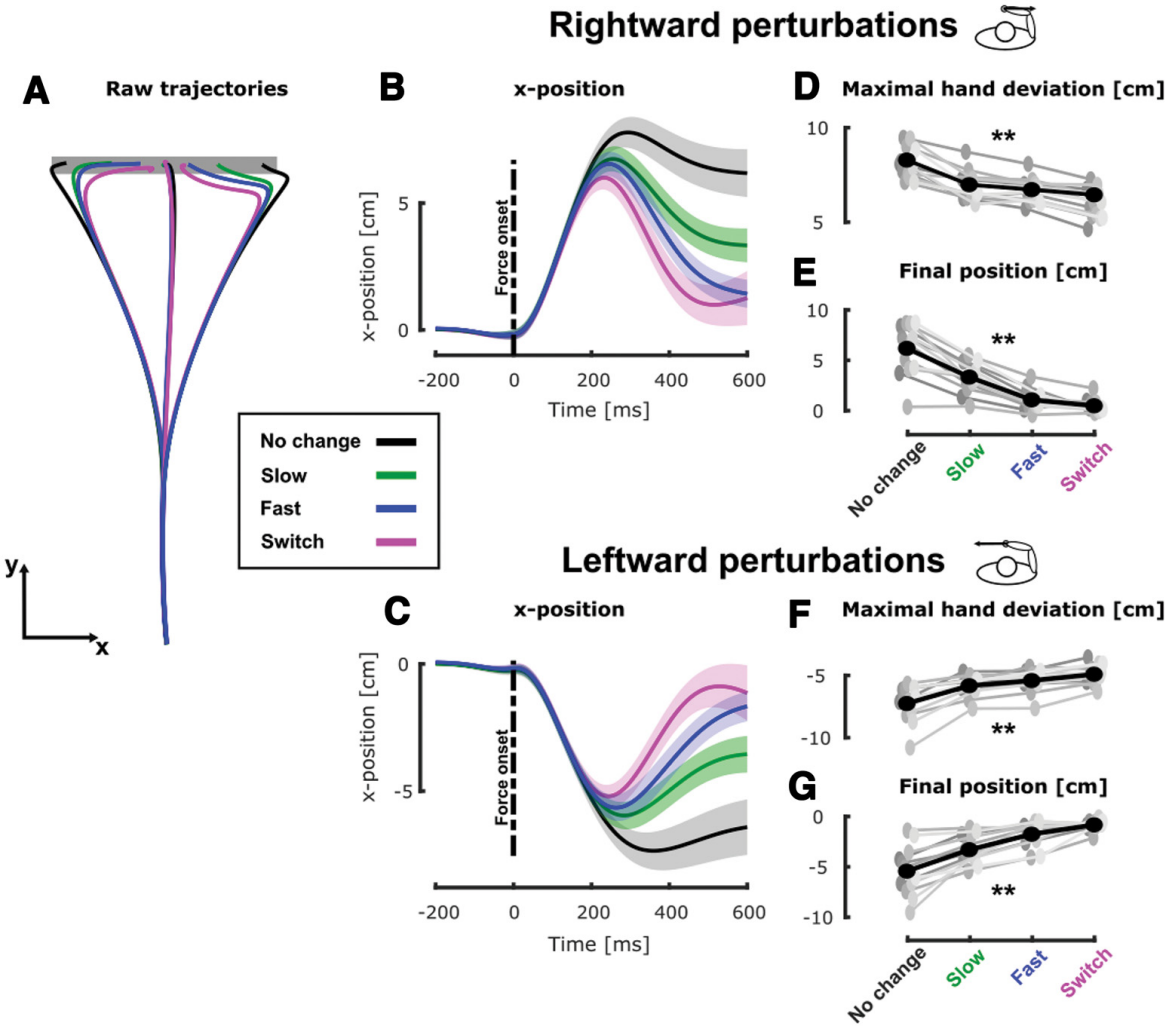
Experiment 1, hand kinematics during movement. ***A***, Group mean of the hand path for unperturbed and perturbed trials in the no change (black), slow (green), fast (blue), and switch (magenta) conditions. ***B***, Group mean and SEM of the x-position of participants’ hand as a function of time (aligned on force onset) for trials perturbed with rightward mechanical perturbations in the four target conditions. The black dashed line represents the onset of the mechanical perturbation. ***C***, Group mean and SEM of the x-position of participants’ hand as a function of time for trials perturbed with leftward mechanical perturbations in the four target conditions. The black dashed line represents the onset of the mechanical perturbation. ***D***, Group mean (black) and individual means (gray) of the maximal hand deviation in presence of a rightward perturbation for the four target conditions. ***E***, Group mean (black) and individual means (gray) of the final position for trials with rightward perturbation for the four target conditions. ***F***, Group mean (black) and individual means (gray) of the maximal hand deviation in presence of a rightward perturbation for the four target conditions. ***G***, Group mean (black) and individual means (gray) of the final position for trials with rightward perturbation for the four target conditions; **p < 0.005.

We observed that the target condition clearly influenced participants’ behavior. Indeed, the mean hand path trajectories in the mechanically perturbed conditions (Fig. 2*A*) differed across conditions. Consistent with our previous findings (De Comite et al., 2021) we observed online adjustments in the behavior in the switch condition (magenta) compared with the no change condition (black). These adjustments consisted of smaller lateral deviations in the switch condition (Fig. 2*B*,*C*, black and magenta traces). Interestingly, the behaviors in the dynamical conditions (slow and fast, green and blue, respectively) differed from both the no change and switch conditions. In order to quantify these differences, we investigated the maximal hand deviation induced by the mechanical perturbations and the final hand position defined as the x-position of the hand as its velocity dropped below 2 cm/s.

The maximal lateral hand deviation induced by rightward mechanical perturbations (Fig. 2*D*) varied significantly across the target conditions. A linear mixed model (see Materials and Methods) revealed a significant effect of target condition ( $\beta_{1}$ = 0.0417 ± 0.0022, *t* = 18.14, *p* < 0.005) on the maximal hand deviation with larger deviations for slower changes in target width. *Post hoc* pairwise analyses revealed that the maximal hand deviation was larger in the no change condition than in slow and fast conditions (slow ${\text{β}}_{\text{1}}$ = −0.006 ± 0.0006, *t* = −10.46, *p* < 0.005, d = 0.60 and fast ${\text{β}}_{\text{1}}$ = −0.007 ± 0.0006, *t* = −12.50, *p* < 0.005, d = 0.71). The hand deviation was larger in these dynamical conditions than it was in the switch condition (fast ${\text{β}}_{\text{1}}$ = −0.0021 ± 0.0008, *t* = −2.56, *p* < 0.005, d = 0.11 and slow ${\text{β}}_{\text{1}}$ = −0.0017 ± 0.0008, *t* = −3.15, *p* < 0.005, d = 0.21). Finally, we even observed that the hand deviation was larger in the slow than in the fast condition ( ${\text{β}}_{\text{1}}$= −0.0013 ± 0.0006, *t* = −2.14, *p* = 0.0319, d = 0.1527). Similar results were observed for leftward mechanical perturbations (see Fig. 2*F*, linear mixed models: ${\text{β}}_{\text{1}}$ = 0.0033 ± 0.0002, *t* = 16.2, *p* < 0.005).

Similarly, we observed that the final hand position along the *x*-axis, computed as the hand position when the total velocity dropped below 2 cm/s, exhibited similar dependency on the target condition. Indeed, a linear mixed model analysis (see Materials and Methods) revealed a significant effect of the target condition ( ${\text{β}}_{\text{1}}$= −0.008 ± 0.0003, *t* = −25.75, *p* < 0.005). As for the maximal hand deviation, *post hoc* pairwise analyses revealed that both dynamical conditions were characterized by less eccentric final hand positions than the no change condition (slow, ${\text{β}}_{\text{1}}$ = −0.014 ± 0.0008, *t* = −16.37, *p* < 0.005, d = 0.80 and fast ${\text{β}}_{\text{1}}$ = −0.025 ± 0.0008, *t* = −28.74, *p* < 0.005, d = 1.27). These final hand positions in the slow condition were more eccentric than the one in the switch condition, no differences were found between the fast and switch conditions (fast ${\text{β}}_{\text{1}}$ = −0.0018 ± 0.0013, *t* = 1.36, *p* = 0.17, d = 0.09 and slow ${\text{β}}_{\text{1}}$ = −0.008 ± 0.0013, *t* = −6.49, *p* < 0.005, d = 0.43). The final hand positions in the slow condition were significantly more eccentric than those in the fast condition ( ${\text{β}}_{\text{1}}$= −0.01 ± 0.0008, *t* = −13.10, *p* < 0.005, d = 0.83). Trials that included a leftward mechanical perturbation (see Fig. 2*G*) contained the same effects (linear mixed models: ${\text{β}}_{\text{1}}$= 0.008 ± 0.0003, *t* = 27.89, *p* < 0.005).

#### Muscle activity

The kinematics results that we reported indicated that participants were able to adjust their control strategy during movements according to dynamical changes in movement goal. They were even able to tune their adjustment to the speed of these dynamical changes. We hypothesized that the stretched EMG activity in Pect. Maj. and Post. Delt. should also depend on the target condition. If such modulation exists in the LL epoch, 50–100 ms following the onset of the mechanical perturbation, it would indicate that the adjustment in behavior did not only reflect changes in voluntary intent but also changes in reflexive responses previously associated with goal-directed state-feedback control (Pruszynski and Scott, 2012; Crevecoeur and Kurtzer, 2018).

**Figure 3.**
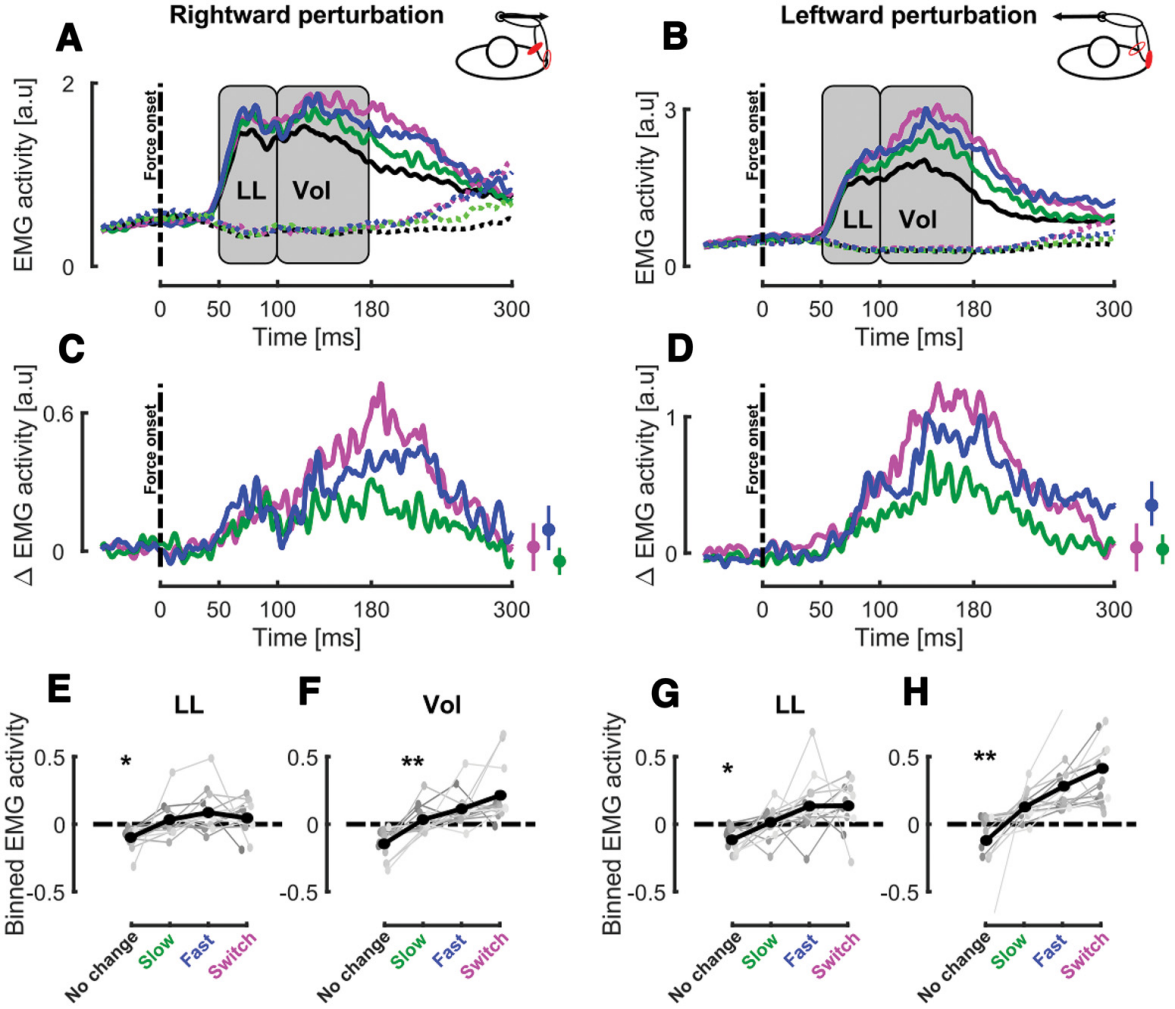
Experiment 1, EMG activity. ***A***, Group mean for the stretched (Pect. Maj., full lines) and shortened (Post. Delt., dashed lines) responses to rightward perturbations in the different target conditions. The gray rectangles represent the LL and VOL epochs where the EMG activity was averaged to perform statistical analyses. The black dashed line represents mechanical perturbation onset and the time axis is aligned with force onset. ***B***, Group mean for the stretched (Post. Delt., full lines) and shortened (Pect. Maj., dashed lines) responses to leftward perturbations in the different target conditions. ***C***, Group mean of target-specific EMG responses to perturbation for Pect. Maj. in the presence of rightward perturbation for the switch (magenta), fast (blue), and slow (green) target conditions. Time axis is aligned with force onset. The small insets represent the mean and SEM target-specific EMG responses at the end of the movement. ***D***, Group mean of target-specific EMG responses to perturbation for Post. Delt. in the presence of leftward perturbation for the switch (magenta), fast (blue), and slow (green) target conditions. Time axis is aligned with force onset. The small insets represent the mean and SEM target-specific EMG responses at the end of the movement. ***E***, ***F***, Group mean (black) and individual means (gray) of the binned EMG activity in the LL (***E***) and VOL (***F***) time windows for Pect. Maj. in the presence of rightward perturbations for the different target conditions. ***G***, ***H***, Group mean (black) and individual means (gray) of the binned EMG activity in the LL (***G***) and VOL (***H***) time windows for Post. Delt. in the presence of leftward perturbations for the different target conditions; *p < 0.05, **p < 0.005.

We observed that the target condition modulated the EMG activity of the muscles stretched by the mechanical perturbation. Figure 3*A*,*B* represent the mean EMG activities collapsed across participants for trials perturbed by rightward or leftward perturbation in all target conditions in the stretched (full lines) and shortened muscles (dashed lines). Visual inspection of target specific responses for the stretched muscles, obtained by subtracting the no change condition, confirmed this modulation of the EMG response (see Fig. 3*C*,*D*, respectively, for Pect. Maj. and Post. Delt.). In order to characterize this modulation, the EMG activity of the stretched muscle was averaged in the LL (50–100 ms after force onset) and VOL time epochs (100–180 ms after force onset) for each perturbation direction. The deviations from the mean activity in these time bins are reported in Figure 3*E*,*F* for stretched Pect. Maj. in the LL and VOL windows at population (black) and individual (gray) levels.

Strikingly, we observed a significant effect of target condition on the modulation of the Pect. Maj. response in the LL (linear mixed models: ${\text{β}}_{\text{1}}$ = −0.029 ± 0.005, *t* = −5.70, *p* < 0.005) and VOL window (linear mixed models: ${\text{β}}_{\text{1}}$ = −0.060 ± 0.00495, *t* = −12 024, *p* < 0.005), respectively, represented in Figure 3*E*,*F*. These negative values indicated larger responses for faster changes in target width. To further investigate these differences, we performed pairwise *post hoc* comparisons between the different target conditions using linear mixed models (see Materials and Methods). In the LL window, we did not observe any difference between the different dynamical conditions (switch/fast ${\text{β}}_{\text{1}}$ = 0.02 ± 0.021, *t* = 0.96, *p* = 0.33, d = 0.05, switch/slow $\beta_{1}$ = −0.0038 ± 0.013, *t* = −0.29, *p* = 0.77, d = 0.015 and slow/fast $\beta_{1}$ = −0.0525 ± 0.0419, *t* = −1.25, *p* = 0.21, d = 0.064), although they all differed from the no change condition (*p* < 0.005 for all conditions). However, these pairwise comparisons revealed significant differences in the VOL time window between the dynamical conditions (switch/fast $\beta_{1}$ = −0.05 ± 0.019, *t* = −2.50, *p* = 0.012, d = 0.11, switch/slow $\beta_{1}$ = −0.059 ± 0.013, *t* = −4.35, *p* < 0.005, d = 0.21 and slow/fast $\beta_{1}$ = −0.079 ± 0.04, *t* = −1.96, *p* = 0.048, d = 0.1).

The same modulation of the EMG activity with the target condition was observed in Post. Delt. for both LL (mixed models: $\beta_{1}$ = −0.046 ± 0.007, *t* = −6.42, *p* < 0.005; Fig. 3*G*) and VOL time epochs (mixed models: $\beta_{1}$ = −0.015 ± 0.008, *t* = −18.66, *p* < 0.005; Fig. 3*F*) when stretched by leftward perturbation. Interestingly, the pairwise *post hoc* comparisons revealed significant differences between the dynamical conditions in both the LL (switch/fast $\beta_{1}$ = −0.005 ± 0.019, *t* = −0.03, *p* = 0.97, d = 0.002, switch/slow $\beta_{1}$ = −0.03 ± 0.012, *t* = −2.336, *p* = 0.019, d = 0.14, and slow/fast $\beta_{1}$ = −0.1193 ± 0.057, *t* = −2.09, *p* = 0.036, d = 0.12) and the VOL time window (switch/fast $\beta_{1}$ = −0.072 ± 0.021, *t* = −3.35, *p* < 0.005, d = 0.14, switch/slow $\beta_{1}$ = −0.12 ± 0.016, *t* = −7.33, *p* < 0.005, d = 0.33 and slow/fast $\beta_{1}$ = −0.25 ± 0.058, *t* = −4.33, *p* < 0.005, d = 0.19). These differences indicated that both reflexive and voluntary responses were modulated by the dynamical change in target width, and suggest that they were even tuned to the rate of change in target width. The significance of the *post hoc* effect between the switch/fast conditions in the voluntary epochs rules out the possibility that participants only used the predicted final target width to modulate their behavior.

Altogether, these results indicate that participants adjusted their behavior during movements in response to dynamical changes in target shape. Indeed, we showed that the hand deviation induced by the mechanical perturbations was different in the dynamical (slow and fast) and in the static conditions (no change and switch). Moreover, we reported larger hand deviation for the slow than for the fast condition: indicating that the rate of change in target width was integrated in the control strategy. The differences observed in acceleration profiles and EMG correlates confirmed this finding. The sensitivity of the online adjustments of control policy to dynamical changes and speed of changes suggest the existence of a mechanism able to finely tune to control strategies within movement.

### Experiment 2

**Figure 4.**
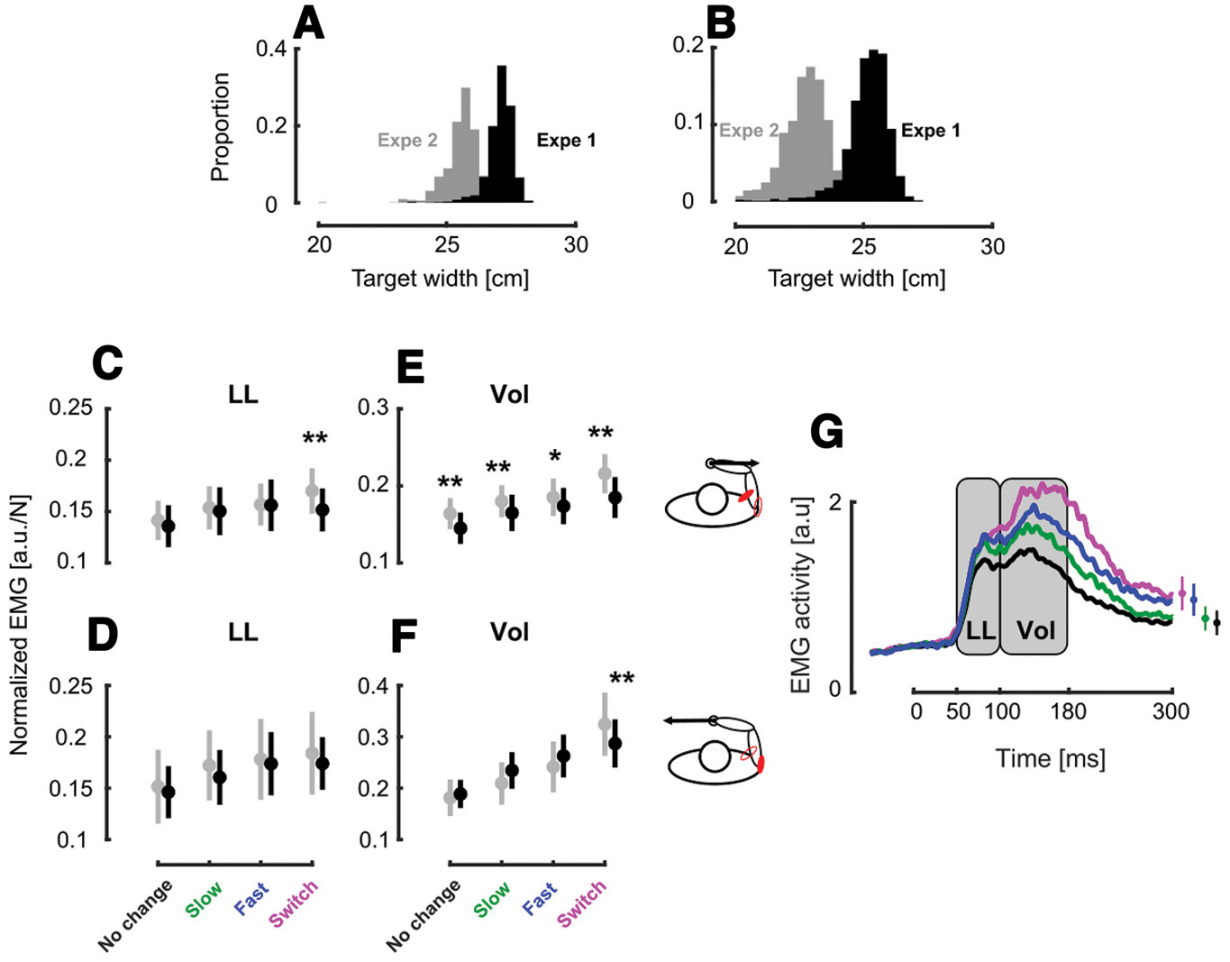
Normalized EMG across experiments. ***A***, Distribution histogram of the target width at perturbation onset in the slow target condition in experiments 1 (black) and 2 (gray). ***B***, Distribution histogram of the target width at force onset in the fast target condition in experiments 1 (black) and 2 (gray). ***C***, Group mean and SEM collapsed across participants of the normalized Pect. Maj. EMG activities in the LL time window in experiments 1 (black) and 2 (gray) across conditions in presence of rightward perturbations. ***D***, Group mean and SEM collapsed across participants of the normalized Post. Delt. EMG activities in the LL time window in experiments 1 (black) and 2 (gray) across conditions in presence of leftward perturbations. ***E***, Group mean and SEM collapsed across participants of the normalized Pect. Maj. EMG activities in the VOL time window in experiments 1 (black) and 2 (gray) across conditions in presence of rightward perturbations. ***F***, Group mean and SEM collapsed across participants of the normalized Post. Delt. EMG activities in the VOL time window in experiments 1 (black) and 2 (gray) across conditions in presence of leftward perturbations. ***G***, Group mean across participants, experiments and muscles of the stretched muscle activity for the different target conditions. The gray rectangles represent the LL and VOL time epochs. The time axis is aligned on force onset and the insets at the right of the panel represent the mean and SEM of the stretched muscles activity at the end of movement; *p < 0.05, **p < 0.005.

Although there was, in experiment 1, a significant effect in the LL window, the pairwise comparisons did not allow to conclude that the modulation was as gradual as in the VOL epoch. We designed experiment 2 to test the possibility that the shallower modulation in the LL, compared with that in the VOL epoch (see Fig. 4 panels E vs F and G vs H), was because of a too short delay between the onset of visual changes and the mechanical perturbation, which could therefore leave too little time to develop a clear modulation in the LL epoch. In experiment 2, the onset of the visual perturbation was similar as in experiment 1 but that of the mechanical perturbations occurred later (150 ms after the visual onset instead of 100 ms in experiment 1) which allowed more time to adjust control policies as a delay of 150 ms was previously reported between the onset of change in target width and changes in control (De Comite et al., 2021).

The impact of the different target conditions on the behavior was qualitatively similar to that of experiment 1 described in Figure 2. We then investigated the modulation of the EMG activity during experiment 2 in the LL and VOL epochs with the same linear mixed model as in experiment 1. We observed significant modulation in both the LL (Pect. Maj.: $\beta_{1}$ = −0.021 ± 0.04, *t* = −4.43, *p* < 0.005 and Post. Delt.: $\beta_{1}$ = −0.014 ± 0.006, *t* = −2.09, *p* = 0.036) and VOL (Pect. Maj.: $\beta_{1}$ = −0.041 ± 0.006, *t* = −6.17, *p* < 0.005 and Post. Delt.: $\beta_{1}$ = −0.062 ± 0.011, *t* = −5.83, *p* < 0.005) time epochs during this control experiment. Similar to what was found in experiment 1, we observed a shallower modulation in the LL time epoch than in the VOL epoch indicating that the design of experiment 1 did not unintentionally reduce the modulation of the response in the LL time epoch.

We investigated whether the differences in responses observed between the fast and the slow conditions result from differences in rates of change in target width or from the instantaneous target width at perturbation onset, by comparing the normalized EMG responses observed in Experiments 1 and 2. If the hypothesis whereby these modulations of the feedback responses are mediated by the width of the target at perturbation onset holds, we should observe larger responses in experiment 2 as the mechanical perturbations were triggered later resulting in smaller target width at perturbation onset (see Fig. 4*A*,*B*). To test this hypothesis, we grouped the normalized stretch muscle activities, binned in the LL and VOL time epochs, from both experiment and investigated a potential effect of the experiment (see Materials and Methods). We did not observe any differences between the normalized responses of experiments 1 and 2, neither in the LL epoch ( $\beta_{2}=0.12\pm 0.08$, *t* = 1.58, *p* = 0.1134; Fig. 4*D*) nor in the VOL epoch ( $\beta_{2}=0.17\pm 0.11$, *t* = 1.43, *p* = 0.1516; Fig. 4*F*). Since we did not observe differences between the feedback responses across the two experiments, we decided to pool these responses to gain a more robust statistical description of the main effect in the LL and VOL time windows. We grouped the muscle activity of the stretched muscles from both experiments (Fig. 4*G* for the mean traces) and used linear mixed models that considered target conditions and experiments as fixed factors (see Materials and Methods). We found a main effect of the target condition in both LL ( $\beta_{1}$= −0.03 ± 0.003, *t* = −9.62, *p* < 0.005) and VOL ( $\beta_{1}$= −0.09 ± 0.003, *t* = −25.61, *p* < 0.005) epochs. *Post hoc* pairwise comparisons performed between conditions in these two time epochs also reported differences demonstrating larger feedback responses for more constrained movements (LL: switch vs fast $\beta_{1}$ = −0.024 ± 0.029, t = −0.89, *p* = 0.39, d = 0.02 switch vs slow $\beta_{1}$ = −0.039 ± 0.013, *t* = −2.80, *p* < 0.005, d = 0.10 and fast vs slow $\beta_{1}$ = −0.063 ± 0.028, *t* = −2.24, *p* = 0.005, d = 0.07 VOL: switch vs fast $\beta_{1}$ = −0.24 ± 0.035, *t* = −7.04, *p* < 0.005, d = 0.35, switch vs slow $\beta_{1}$ = −0.19 ± 0.017, *t* = −11.504, *p* < 0.005, d = 0.21 and fast vs slow $\beta_{1}$ = −0.15 ± 0.031, *t* = −4.89, *p* < 0.005, d = 0.14).

This second experiment revealed that the modulation of EMG activity in the LL epoch was small but robust and reproducible. We also found across the two experiments, for which the target width at perturbation onset was different, that the responses were very similar. Observe that the perturbation in experiment 2 were triggered a bit later, which potentially increase the response gains (Poscente et al., 2021). Thus, this effect should add to a potential sensitivity to target width. Nevertheless, we found essentially similar normalized EMG despite (slightly) later occurrence and smaller instantaneous width. This result suggests that the underlying neural pathways may consider the speed or rate of change of target width, which is clearly consistent with our hypothesis that continuous change in task parameters modulate control gains dynamically.

### Differences between the first and last trials in dynamical conditions

**Figure 5.**
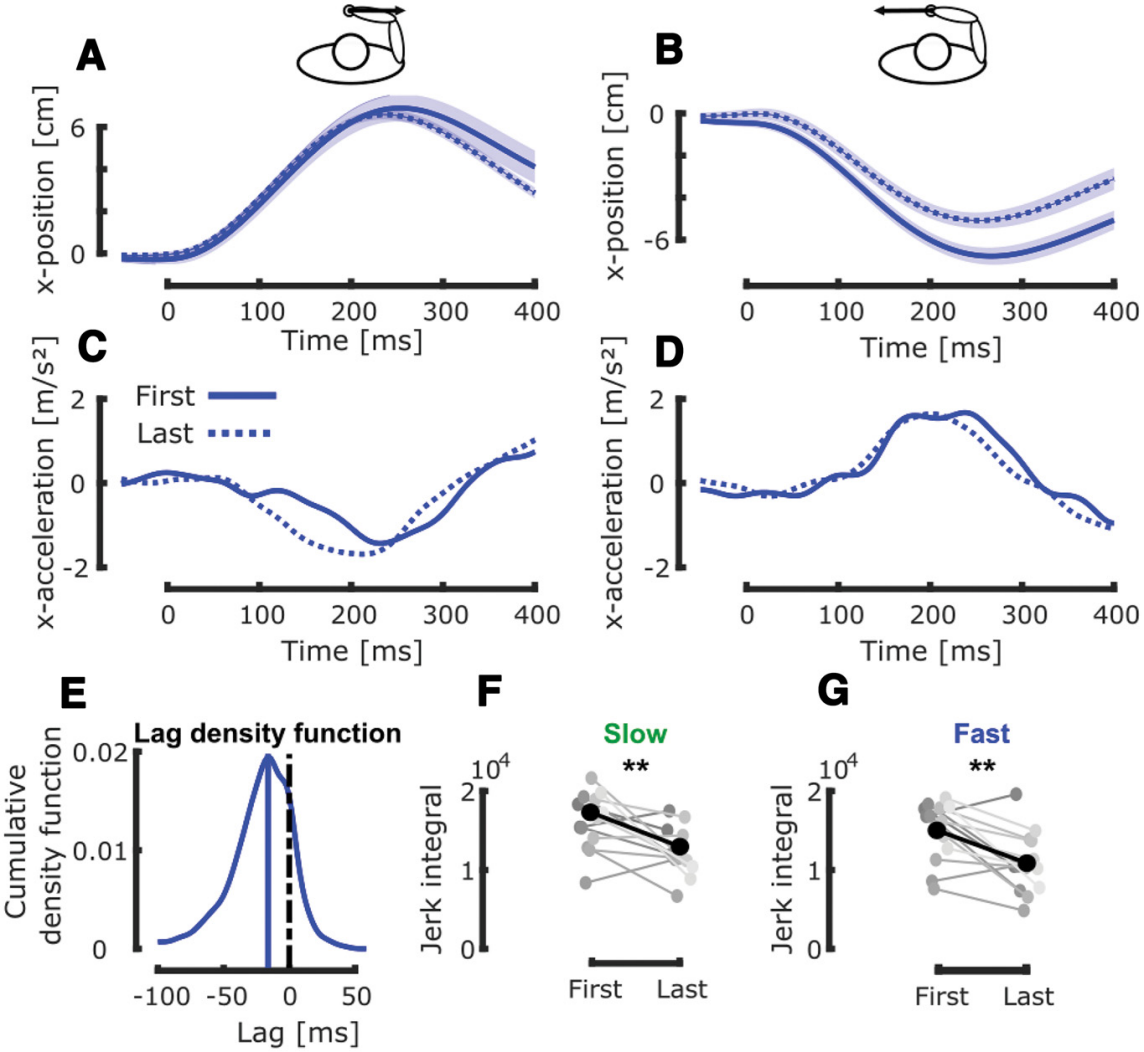
Between trials analyses. ***A***, Group mean and SEM collapsed across participants of the first (full line) and last (dashed line) trials in the fast condition with rightward mechanical perturbation. Time axis is aligned on force onset. ***B***, Group mean and SEM collapsed across participants of the first (full line) and last (dashed line) trials in the fast condition with leftward mechanical perturbation. The black dashed line corresponds to the difference between the first and last trials. Time axis is aligned on force onset. ***C***, Group mean of the acceleration profiles of the first (full line) and last (dashed line) trials in the fast condition with rightward mechanical perturbation. Time axis is aligned with force onset. ***D***, Group mean of the acceleration profiles of the first (full line) and last (dashed line) trias in the fast condition with leftward mechanical perturbation. Time axis is aligned with force onset. ***E***, Cumulative density function of the lag between the last and first acceleration profiles for both perturbation directions. The blue vertical line corresponds to the median value. ***F***, Group mean (black) and individual means (gray) of the integral of the absolute value of the jerk for the first and last trials in the fast target condition. ***G***, Group mean (black) and individual means (gray) of the integral of the absolute value of the jerk for the first and last trials in the slow target condition; **p < 0.005.

Interestingly, we observed that participant’s behavior during the fast and slow conditions changed across blocks. Figure 5*A*,*B* represents the mean and SEM of the position along the *x*-axis for the first (full line) and last trials (dashed line) in the fast condition for rightward and leftward mechanical perturbations, respectively. We observed that these first and last trials differed and decided to take a look at their acceleration profiles to quantify these differences. The corresponding acceleration profiles are represented in Figure 5*C*,*D*. We observed a consistent and significant lag of the last trial with respect to the first one. This lag was computed by taking the median of the lags distribution that was obtained from the maximal values of the cross-correlation between the first and last *fast* trials of the fourteen subjects with 10,000 bootstrap samples (see Materials and Methods). The resulting distribution of this lag, obtained through this resampling method is represented in Figure 5*E*. This method revealed a median lag of –18 ms (Fig. 5*E*, blue vertical line) that was significantly smaller than zero (signrank test z = −32.18, *p* < 0.005).

The first and last trials of each dynamical condition also differed in the smoothness of their acceleration profile as shown in Figure 5*C*,*D* for the fast condition. This difference in smoothness was quantified by comparing the integral of the absolute values of the derivatives of these acceleration profiles: the jerk. We reported in Figure 5*F*,*G*, these integrals for all participants in the slow and fast conditions, respectively. In the fast condition, the final state was less jerky than the first one as reported by a signrank test (z = −2.835, *p* < 0.005). Similar results were obtained in the slow condition (signrank test z = −3.19, *p* < 0.005) indicating an increase in the smoothness of the acceleration profiles.

Thus, there were measurable behavioral changes that could be related to practice; however, they did not interfere with the interaction between target condition and behavior. Indeed, we still observed a significant modulation of the EMG activity in both LL (linear mixed models, $\beta_{1}$ = −0.041 ± 0.009, *t* = −4.21, *p* < 0.005) and VOL epochs (linear mixed models, $\beta_{1}$ = −0.144 ± 0.014, *t* = −10.11, *p* < 0.005) when we only considered the last twelve trials of each dynamical condition for all participants.

## Discussion

We investigated how humans responded to continuous changes in target width during reaching. More specifically, we studied participants’ behavior as they were reaching to a target, initially represented as a wide rectangle, with time varying width. We observed that the way participants responded to unexpected mechanical perturbations depended on the target condition and specifically on the rate of change in target width during movement. This demonstrated that the control policies used to perform reaching movements were adjusted online to the specific change in target width, which captures participants’ ability to continuously track and respond to task parameters during movement.

Here, we leveraged an experimental paradigm developed in a previous work (De Comite et al., 2021), consisting of abrupt changes in target structure within movements, to dynamically alter the task constraints and investigate whether participants’ control policies were adjusted online. This paradigm exploits the minimum intervention principle (Todorov and Jordan, 2002), which states that participants only correct deviations that interfere with the task success during reaching movements. This means that participants exploit the target redundancy when available, even in the absence of perturbations (Scholz et al., 2000; Vetter et al., 2002; Berret et al., 2011; Knill et al., 2011; Nashed et al., 2012; Togo et al., 2017). The observed behavior and the feedback responses to mechanical perturbations confirmed that these control policies were adjusted during movement. Indeed, we reported modulations induced by the different dynamical changes in target width, corresponding to different alterations of the cost-function and that this mechanism considered the rate of change in target width. In our view, these results demonstrate the existence of a mechanism that adjusts the control policy during movement thanks to a continuous tracking of target width. In the present study, this task-specific adjustment in control relied on visual processing of task-parameters and impacted LL and VOL responses to the mechanical perturbations.

**Figure 6.**
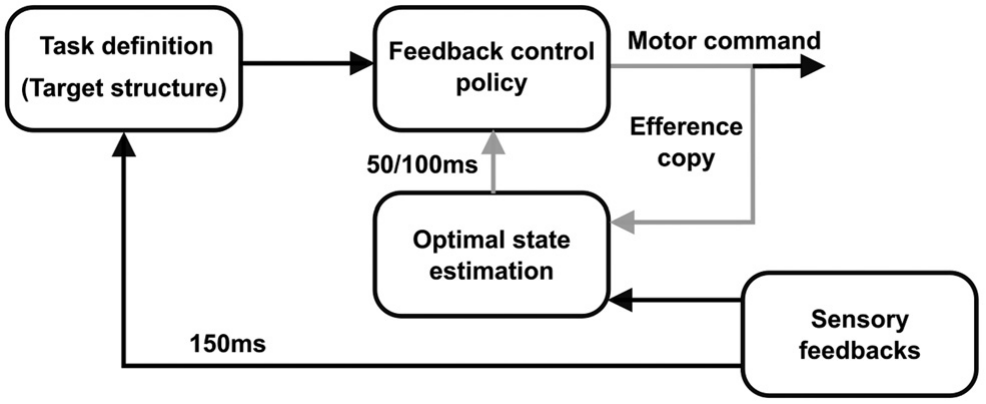
Modified goal-directed feedback control architecture. The inner loop (in gray), combining sensory feedbacks and efference copy is the one responsible for the goal-directed feedback responses observed behaviorally. The latency of this inner loop is 50–100 ms depending on the sensory modalities involved. The outer feedback loop (in black) that modifies the task definition and the feedback control policy captures the online adjustments in control policy elicited by continuous alteration of the target structure during movement.

We must emphasize a critical difference between a feedback response to an external perturbation and the results that we highlighted here. In standard perturbation paradigms, visual or mechanical events alter the state of the system, including limb and target position, velocity, and higher order derivatives. These perturbation paradigms allowed showing that the control policy used to perform movement is tuned to the task-goal as demonstrated by the goal-dependent characteristics of the feedback responses to disturbances (Knill et al., 2011; Nashed et al., 2012; Sarlegna and Mutha, 2015; Keyser et al., 2017; Lowrey et al., 2017). These feedback responses are defined by rapid feedback loops (Fig. 6, inner loop, gray) whose latencies depend on the sensory modalities involved (Franklin and Wolpert, 2008; Knill et al., 2011; Pruszynski and Scott, 2012; Scott, 2016). In the case of mechanical disturbances applied to the limb, this inner feedback loop is mediated by LL feedback pathways that have a latency of 50 ms (Pruszynski and Scott, 2012). Here, we probed not only the feedback responses to changes in the state of the system, but also the change in the controller itself in response to dynamic changes in task parameters during movement. Taken in the context of OFC (Todorov and Jordan, 2002; Scott, 2004; Shadmehr and Krakauer, 2008), the task-parameters (such as target width) define the cost-function and the control law (Nashed et al., 2012), which is derived from these cost parameters. We demonstrated that this selection of the control policy based on the task parameters is itself continuous and must be considered in closed loop control models of human reaching movements (Fig. 6, outer loop, black). The latency of this outer loop was ∼150 ms as reported in previous work (De Comite et al., 2021), here we used this number to design the task, and highlighted that indeed dynamic changes in the task parameters have an impact in the LL feedback pathways.

This interpretation implies a possible overlap of movement planning and execution, as participants may alter their motor plan during an ongoing movement. Such overlap of planning and execution has been suggested in studies reporting that reaction times before movement initiation could be shortened at the price of a reduced accuracy (Haith et al., 2016; Orban de Xivry et al., 2017). More recently, it has been suggested that such overlap of planning and execution could occur during movement in presence of visual perturbations (Dimitriou et al., 2013; Česonis and Franklin, 2020, 2021). However, these results could also be explained by other mechanisms such as an infinite horizon controller (Li et al., 2018). In our previous study, we provided clear evidence for this overlap of movement planning and execution in presence of perturbations that altered the cost-function from which the control policy is derived (De Comite et al., 2021). This overlap is also necessary to explain the present results as participants have to continuously adjust their control strategy in response to the change in target width.

These continuous adjustments of control policy are reminiscent of the theoretical framework of model predictive control (for review, see Lee, 2011). This framework posits that the control policy is continuously adjusted during movement to integrate any change in the cost-function or in the environmental context. An alternative hypothesis to explain these online adjustments in control policy is that participants switched between several prespecified control policies. A similar process was suggested to account for the selection of the most appropriate strategy specified in parallel (Chapman et al., 2010; Gallivan et al., 2016, 2017; Wong and Haith, 2017) but was questioned and compared with a single optimal intermediate motor plan (Haith et al., 2015; Alhussein and Smith, 2021). Our experimental paradigm differed as multiple options were never presented at the same time and changes between targets occurred within movements.

We favor an interpretation that assumes dynamical adjustments of the control strategy although we cannot formally rule out the possibility of discrete switches between different predefined controllers. However, there are observations that do plead for a continuous and dynamic monitoring. First, we observed that the first slow and fast trials where different, although participants had not encountered these conditions during training and therefore could not have acquired a controller tuned to these specific conditions at that time. This was observed despite the fact that these first trials were jerkier than the later ones, which could indicate that even when participants had not familiarized with the dynamical conditions, they seemed to exploit well the outer feedback loop as input to the controller relative to target structure (Fig. 6, outer loop). One caveat to the hypothesis of continuous monitoring was that the normalized feedback responses across experiments 1 and 2 (Fig. 4) did not change much with longer viewing time, which suggests that there may be constraints on the amount of modulation that can take place. Nevertheless, this absence of modulation of the feedback responses within movement must be interpreted with caution because other factors such as time or urgency also modulate these feedback responses within movement (Crevecoeur et al., 2013; Dimitriou et al., 2013; Poscente et al., 2021).

A parallel can be drawn between the present study and a series of studies that reported within-trials tuning of feedback corrections when exposed to velocity-dependent force fields randomly (Crevecoeur et al., 2020a,b; Mathew et al., 2020; Mathew and Crevecoeur, 2021). It proposed that continuous tracking of model parameter also happens during movement, suggesting that adaptation to an altered plant dynamics also happens online (Crevecoeur et al., 2020b). In the present study, we reported the online tracking of cost parameters that define the movement goal. Interestingly, besides the conceptual similarity between these two processes linked to online evaluation of task or dynamical parameters, they were associated to different latencies: ∼150 ms for updating the control policies following changes in movement goal (based on the present and on De Comite et al., 2021), while a latency of ∼250 ms was associated with the online tuning of the feedback controller (Crevecoeur et al., 2020b). It is therefore conceivable that they engage dissociable neural operations that remain to be investigated.

An interesting question is to determine which neural substrates are involved in the online modulation of the reaching controller. Sensorimotor control is mostly supported by multiple cortical areas, the basal ganglia, and the cerebellum (Shadmehr and Krakauer, 2008; Scott, 2016; Haar and Donchin, 2020). We identify two neural pathways that likely underlie the online changes in behavior documented in the present study. The first is that the parametric feedback controller supported by the LL feedback (Pruszynski and Scott, 2012; Crevecoeur and Kurtzer, 2018) is modulated online. Such modulation must have occurred based on visual input which has a fast route to the network supporting LL responses through associative areas in the parietal cortex (Cross et al., 2021). The second pathway that is likely involved is related to the definition of the task demands, which includes the basal ganglia known to represent motor costs (Mazzoni et al., 2007; Shadmehr and Krakauer, 2008). Since our task paradigm altered the motor costs by modulating the target width, it is conceivable that the adjustment in control policy depended on a signal originating from the basal ganglia inducing the change of controller or a selection of a different controller. Note that this cannot happen independently of the visual input, and thus any interaction between rapid feedback pathways conveying information about the target structure, and selection of controllers based on a representation of motor costs in basal ganglia may support the observed change in behavior.

To sum up, we reported here that humans are able to dynamically adjust their control policy when they experience a dynamical change in task demands. These findings highlight the existence of a continuous monitoring of task-related parameters which supports dynamic changes in online feedback control.

## Synthesis

Reviewing Editor: Michael Michaelides, NIDA-NIH Decisions are customarily a result of the Reviewing Editor and the peer reviewers coming together and discussing their recommendations until a consensus is reached. When revisions are invited, a fact-based synthesis statement explaining their decision and outlining what is needed to prepare a revision will be listed below. The following reviewer(s) agreed to reveal their identity: Tarkeshwar Singh, Opher Donchin. Note: If this manuscript was transferred from JNeurosci and a decision was made to accept the manuscript without peer review, a brief statement to this effect will instead be what is listed below.

----------------------------------------

Reviewer 1

The authors conducted a motor control study here and investigated how changing the width of a target during a reaching movement affected mechanosensory feedback gains. These gains were measured in the long-latency and voluntary control epochs. The experimental task fits well within the framework of optimal feedback control and is a reasonable, well-designed, and well-executed extension of the prior work published by these authors. Overall, the work is well done, and I really liked the clever design of this study. But there are some aspects of the work that could be explained more clearly. My major concerns are that the authors have not justified well why a second experiment was done and why certain analyses were performed? In addition, I found the Discussion substantive and quite detailed, but lacking the breadth that could provide motor control scientists new ideas and hypotheses to build on this work. I sincerely request the authors to explain their results within the broader framework of motor control literature instead of narrowly focusing on optimal feedback control.

Major comments:

Introduction

1) I am not clear why the second experiment was done. The rationale for this should be explained clearly and succinctly in the Introduction. I am also sorry to say that the rationale for this experiment currently is neither strong nor coherent.

Methods

1) Line 200: “In order to investigate the lag between the first and last trials in the dynamical conditions, we used a cross-correlation analysis applied on resampled data.” I am not clear why this analysis is being done. Could you provide a clearer rationale, please? Also, what do you mean by “dynamical conditions”? Which blocks of the first and last trials are being referred to here? If you are trying to look at some form of learning, then that should be introduced in the Introduction.

Results

1) Line 296: “tuned to the rate of change in target width”. Maybe I am not following the logic here. Based on what you have said in Lines 139-141, I would expect that this strong statement will only be supported if the post-hoc tests between switch/slow and slow/fast were significant, but switch/fast were not significant. You controlled the speed of the fast condition to match the slowest correct movement in the switch condition. I think you need to elaborate both why you chose to do what you did in lines 139-141 and how these post-hoc tests support this strong statement.

2) Lines 311: Again, I am not clear very Exp. 2 was done. Is it based on Reviewer feedback from a previous iteration of review in a different or same journal? Regardless, the authors could clarify the intent better and make the two experiments flow better. They bring up “shallower modulation in LL epoch”. This has not been introduced in the Introduction. It is not clear to me why this shallow versus steep modulation is important. Also, am not clear where the “50 ms” in line 313 comes from. They talk about it briefly in the Discussion, but are you assuming that readers are all familiar with the latencies in play for short-latency and long-latency feedback?

Discussion

1) I think the authors have done a decent and fair job explaining their results within the framework of optimal feedback control. However, the Discussion is written for an engineering journal, and not a neuroscience journal. I strongly urge the authors to make a sincere attempt to relate these findings to: a) likely neural substrates (parietal and premotor interactions) for setting of feedback gains for different conditions; b) other established phenomena in motor control such as Fitts’ Law; and c) multisensory (visuo-proprioceptive) integration. Their well-conceived and executed experimental framework can bridge a lot of disparate experimental findings and provide the bases for generation of new hypothesis and experimental paradigms. However, for that we need the authors to do the heavy duty thinking and make a sincere attempt to explain their results within the broader context of the field of motor control, instead of just staying in their comfort zone of optimal feedback control.

Minor comments:

Introduction

1) Line 60-62: I think you can phrase it better than this statement. So many theories claim that the nervous system optimizes a cost function.

2) Line 89: “wide versus narrow” this comes out of nowhere. What are readers supposed to make of this? Can you relate this clearly to what is going to happen to target width so that readers are not left guessing.

Methods

2) Lines 130-153: Can the authors make a table or a diagram to show the overall experimental design? This is verbose and frankly not very clear.

3) Line 144: I understand that the trials were interleaved but when the participants initiated the reaching movements towards the target, did they have any clue whether the condition was switch, fast or slow? Or would they have to figure that out themselves in the first 6 cm (or 8 cm for Exp 2)?

4) Line 150: why were there two more trials for each target condition during mechanical perturbation?

5) Line 194: Could you provide more details for how the statistical model was created? How were individual trials included in the model? Did you use the mean for each condition in the model? Please provide details so other researchers can replicate your analyses.

Results

3) Figure 2: Could you add legends to panels 2a-c?

4) Line 227: for p values, you would typically report either p<0.05, p<0.01 or p<0.001 or the exact p-value. I would suggest reporting the exact p-value.

5) Line 228: “consistent with our"

6) Line 284: same comment for p values as my previous comment

7) Lines 281-287: I think these tests and the many following tests could be better represented in a tabular format.

8) Line 288: I just noticed the abbreviation PD for posterior deltoid. Could you please use Post Delt or something else? I think PD should be reserved for Parkinson’s Disease.

9) Line 292-295: same tabular format would perhaps be better

10) Line 309: shallower modulation? Could you refer to the figure and the panel so it is easy to follow the authors’ thoughts.

11) Line 312: Which “one” of the mechanical perturbations? I thought there is only one mechanical perturbation in each trial.

12) Line 321: “reduce"

13) Line 323: please label the figures. It is difficult to keep track of the figures without the numbers displayed on the figures.

14) Line 331 and 335: Now you are referring to muscles sometimes as PM and sometimes as Pectoralis Major. I think abbreviations like Pect Maj and Post. Delt are better because it does not force the reader to think what is being referred to here.

15) Line 342: Do you mean 4G?

16) Line 384: Why did you choose only the last 12?

Discussion

1) Line 386: “task parameters” this is vague. Please elaborate briefly and clearly what was manipulated (target width) and the intent behind those manipulations?

2) Line 401: Again, you have a specific experiment. You are modulating the width of the target at different rates. Using a generic phrase like “task parameters” just confuses the reader. Please be more specific.

3) Line 409: Could you please put the inner loop in a different colour?

Reviewer 2

The authors explore a task where participants make movements to a target that starts wide but can the switch to narrow or else shrink to narrow at one of two different speeds. Because there movement is occasionally perturbed to the right or left, participants must make an ongoing correction to hit the target and this correction increases in conditions where the target is narrower when it is hit. That is, there is least correction when the target stays wide, more for a slowly changing target, more even for a quickly changing target, and the most for a target that switches to a narrow target. The authors interpret this as an online adjustment of the control policy driven dynamically by changes in the conditions.

This work follows up a similar study which only included the no change and switch conditions. The claim of the authors is that the fact that responses change also between the two conditions the target shrinks slowly or quickly shows that the participants are not merely switching between conditions but instead dynamically adjusting control parameters.

I have a number of concerns about this work. They are organized in the order in which I view their importance from most to least.

1. I do not see why adjustments of the control policy are necessary. It seems to me that if target endpoints are represented in the state vector, then a single control poicy would be sufficient. When target width changes, feedback response would automatically increase because the control policy would see a larger gap between current predicted position and target.

2. Even if control policy is adjusted in the different conditions, I do not see how we can tell that the participants rae adjusting it dynamically. They may be identifying which of the 4 conditions they are currently facing (based on assessment of the rate of change) and choosing one of the 4 prepared policies. The authors make this point themselves in lines 438-440 and I did not understand how the lines following addressed this issue. They seem to move over to the question of intermediate strategies rather than addressing the possibility of a rapid switch in control.

3. It was not clear to me what the analysis of EMG adds to the paper. Ultimately, if movements change then EMG must as well. The question that the EMG is supposed to answer could be made clearer. From the discussion and the statistical approach, I came to the conclusion that the changes in EMG where meant to test for the difference between adjustments based on changes in the control strategy (during the LL period of 50-100 ms) and voluntary changes in intent (during the VOL period of 100-180 ms). I’m not at all certain that this interpretation is correct. If so, it might represent a response to my point 1. On the other hand, the changes in the EMG in the LL period are quite small compared to the changes during the VOL period and do not seem to be consistent across conditions. This would suggest that change in movement path is driven largely (if perhaps not entirely) by the EMG changes in the VOL period. Almost all of their behavioral effect is then explained by the VOL period EMG. Again, I am not sure I understand well enough to know how this affects the overall interpretation. Perhaps the authors could make this clearer.

4. I have concerns about the model that was used for assessing significant differences. Throughout the text, the authors report beta1 as a single number, suggesting that the effect of condition is modeled as a regressor rather than as 4 separate conditions. If that is right, this would be a mistake. Since the authors are using fitlme, the key point is that condition in the matlab table needs to be specified as a categorical variable. I may be wrong about what they’ve done, but otherwise I can’t make sense of having a single parameter give the effect size for condition.

5. It is also not clear that from the text that they have properly specified the repeated measures character of the experimental design. That is, the formula between lines 194 and 195 does not seem to explicitly specify that this is a repeated measure design and traditionally this is not how it would be written. This might explain the error bars in Figure 4C-F that seem to me to be suspiciously small.

6. The follow up paired tests between groups seem to be done using a direct paired test instead of a comparison between parameters of the regression. If so, this would also be a mistaken approach. Post hoc comparisons following a regression should be performed on the parameters of the regression (that is looking at contrasts of the dummy variables representing the different groups in the categorical variable for condition). Compounding this, the authors seem to be using a nonparametric (ranksum) paired comparison when the regression was specified as a parmaetric analysis. This choice is not justified and seems aribtrary.

7. The authors should provide effect size and confidence intervals for comparisons between coditions.

8. In the analysis of the EMG, the authors interpret the difference between a significant and a non-signficant result to mean that there is a meaningful difference between the outcomes (such as on line 281 but in other place as well). They also sometimes treat a non-significant result as an indication of lack of effect (such as on line 333). It is important only to draw conclusions following a direct statistical test of the claim being made and not to draw conclusions from lack of significance (unless an explicit test for equivalence or some other clearly stated criterion for equivalence is used).

9. When providing errors in the text, the authors do not make clear if they are standard errors of the parameter or its confidence interval.

10. The authors should add errobar patches like those in Figure 2 wherever it makes sense. This includes Figure 3A-D and Figure 4G.

11. In Figure 3E-H, it is very hard to resolve the data from the different subjects. I recommend using slightly different grayscale or color to allow subjects to be better differentiated and also smaller dots for the markers. In addition, you can jitter the x position of the different subjects slightly to help make them more visible.

12. The figure legends are formatted centered. I think that might be an oversight. Personally, I would find it much easier to review the figures if the figure legends appeared following the figures instead of needing to jump back and forth.

## Author Response

We are extremely grateful to the Reviewers and the Editor for their assessment of our work. The important points they raised helped us to improve the manuscript. Please find here below a point‐to‐ point response to the comments that were made alongside with references to the modifications in the text.

Reviewer 1

The authors conducted a motor control study here and investigated how changing the width of a target during a reaching movement affected mechanosensory feedback gains. These gains were measured in the long‐latency and voluntary control epochs. The experimental task fits well within the framework of optimal feedback control and is a reasonable, well‐designed, and well‐executed extension of the prior work published by these authors. Overall, the work is well done, and I really liked the clever design of this study. But there are some aspects of the work that could be explained more clearly. My major concerns are that the authors have not justified well why a second experiment was done and why certain analyses were performed? In addition, I found the Discussion substantive and quite detailed, but lacking the breadth that could provide motor control scientists new ideas and hypotheses to build on this work. I sincerely request the authors to explain their results within the broader framework of motor control literature instead of narrowly focusing on optimal feedback control.

Major comments:

Introduction

1) I am not clear why the second experiment was done. The rationale for this should be explained clearly and succinctly in the Introduction. I am also sorry to say that the rationale for this experiment currently is neither strong nor coherent.

The aims of Experiment 2 were (i) to reproduce the results of Experiment 1, and (ii) to investigate whether the modulation of feedback responses to the mechanical perturbation depended on the perturbation timing. Indeed, in Experiment 1, there was on average 150ms between the onset of change in target structure (triggered as participants left the home target) and the beginning of the LL epoch, which was the earliest epoch during which we investigated the impact of target condition on the feedback response. This timing corresponded to the delay previously observed to adjust the control policy in response to large salient changes in target width (see (De Comite et al. 2021)). In both experiments of the present study, the differences in target width across conditions were smaller which could have reduced the effect in the early time window.

Equivalently, Experiment 2 also allowed us to determine whether the feedback responses to mechanical perturbations evolved with time within a trial or whether they were constant once the target condition was identified. Combined data from Experiments 1 and 2 informed us that these responses did not change much during movements, suggesting a common adjustment in strategy depending on the rate of change in target width (lines 368‐390).

We added this information to the revised version of the manuscript in the Introduction and Methods sections. The Introduction was changed on lines 91‐93 to emphasize the fact that participants’ adjustments responded to specific changes in target width rather than to the target width at perturbation onset. We also modified the Methods section on lines 160‐162 to describe the rationale for this second Experiment in more detail.

Methods

1) Line 200: “In order to investigate the lag between the first and last trials in the dynamical conditions, we used a cross‐correlation analysis applied on resampled data.” I am not clear why this analysis is being done. Could you provide a clearer rationale, please? Also, what do you mean by “dynamical conditions”?

Which blocks of the first and last trials are being referred to here? If you are trying to look at some form of learning, then that should be introduced in the Introduction.

This analysis was performed to quantify the impact of practice or habituation on behavior in the slow and fast conditions (referred to as dynamical conditions throughout the manuscript). Indeed, participants were not exposed to these conditions during the training blocks (line 149‐150) and only encountered these trials during the experiment. We compared the very first and very last trials of these slow and fast conditions, across the whole experiment and found evidence for learning across trials from the timing of feedback responses. The rationale for this analysis has been clarified in the Methods section (lines 237‐240).

Results

1) Line 296: “tuned to the rate of change in target width”. Maybe I am not following the logic here. Based on what you have said in Lines 139‐141, I would expect that this strong statement will only be supported if the post‐hoc tests between switch/slow and slow/fast were significant, but switch/fast were not significant. You controlled the speed of the fast condition to match the slowest correct movement in the switch condition. I think you need to elaborate both why you chose to do what you did in lines 139‐141 and how these post‐hoc tests support this strong statement. When we pooled data from the agonist muscles in the two experiments we observed a global effect of target condition as well as posthoc differences between the slow and switch conditions during the LL epoch (lines 368‐390). Moreover, we reported posthoc differences between the switch and fast conditions in the stretched LL response of the Posterior Deltoid in Experiment 1 (lines 330‐331). We acknowledge that we did not report differences between the switch and fast conditions in all muscles and experiments but our data suggest that participants’ adjustments were able to discriminate between the slow and fast conditions and, as a consequence, were tuned to the specific rate of change in target width. In addition, we observed similar modulations across experiments where the rates were the same but not the net width at perturbation onset.

We decided to match the final width in the fast condition to assess whether participants used a prediction about the terminal target width, in which case the responses in the switch and fast conditions would be identical (see lines 143‐145). It was clear from our data that these two conditions were not equivalent

2) Lines 311: Again, I am not clear very Exp. 2 was done. Is it based on Reviewer feedback from a previous iteration of review in a different or same journal? Regardless, the authors could clarify the intent better and make the two experiments flow better. They bring up “shallower modulation in LL epoch”. This has not been introduced in the Introduction. It is not clear to me why this shallow versus steep modulation is important. Also, am not clear where the “50 ms” in line 313 comes from. They talk about it briefly in the Discussion, but are you assuming that readers are all familiar with the latencies in play for short‐latency and long‐latency feedback?

Experiment 2 was run to assess reproducibility of the results of Exp. 1 at longer latencies and determine whether participants’ adjustment was tuned to the rate of change in target width or to the target width at perturbation onset (lines 91‐93). The shallower modulation in the LL epoch (compared to the VOL epoch) is captured in Figure 3 E vs F and G vs H. We suspected that our experimental design (namely the 150ms delay between the onset of change in target structure and the beginning of the LL epoch (Pruszynski et al. 2008; Pruszynski and Scott 2012)) could limit the modulation of LL responses. In this case, a longer delay would enable larger modulation of LL responses, which was the purpose of Exp 2.

The data suggested that it was not the case and, moreover, provided some information about the mechanism underlying the change in control. More details about the different sensorimotor delays were added in the revised version of the manuscript so that readers were not assumed to know them (lines 348‐357).

Discussion

1) I think the authors have done a decent and fair job explaining their results within the framework of optimal feedback control. However, the Discussion is written for an engineering journal, and not a neuroscience journal. I strongly urge the authors to make a sincere attempt to relate these findings to: a) likely neural substrates (parietal and premotor interactions) for setting of feedback gains for different conditions; b) other established phenomena in motor control such as Fitts’ Law; and c) multisensory (visuo‐proprioceptive) integration. Their well‐conceived and executed experimental framework can bridge a lot of disparate experimental findings and provide the bases for generation of new hypothesis and experimental paradigms. However, for that we need the authors to do the heavy duty thinking and make a sincere attempt to explain their results within the broader context of the field of motor control, instead of just staying in their comfort zone of optimal feedback control.

We would like to thank the reviewer for giving us the opportunity to explain our results in a broader context, even if this discussion is a bit more speculative and relies on experimental evidence from previous studies. Briefly we identified two possible neural pathways underlying the observed behavior. The first pathway contains the basal ganglia which are known for their role in the task definition as they are sensitive to motor cost (Mazzoni et al. 2007), which is altered when we modified the target width.

The other hypothesis is that the parametric feedback controller characterized by the long latency responses is modulated by the visual input through associative areas of the parietal cortex. This point is discussed in more detail in a new discussion paragraph (lines 520‐535).

Minor comments:

Introduction

1) Line 60‐62: I think you can phrase it better than this statement. So many theories claim that the nervous system optimizes a cost function. We clarified it by specifying what exactly this control policy is doing (lines 60‐64).

2) Line 89: “wide versus narrow” this comes out of nowhere. What are readers supposed to make of this? Can you relate this clearly to what is going to happen to target width so that readers are not left guessing.

This has been clarified in the revised version of the manuscript to indicate that it refers to the target width (lines 87‐88).

Methods

2) Lines 130‐153: Can the authors make a table or a diagram to show the overall experimental design?

This is verbose and frankly not very clear.

A table recapitulating the trial distribution within the two experiments has been added to the revised version of the manuscript (see lines 157‐158). 3) Line 144: I understand that the trials were interleaved but when the participants initiated the reaching movements towards the target, did they have any clue whether the condition was switch, fast or slow? Or would they have to figure that out themselves in the first 6 cm (or 8 cm for Exp 2)?

There was no clue about the target condition prior to movement, participants had to figure it out in the first 6 or 8 cm. This has been clarified in the revised version of the manuscript (lines 136‐137).

4) Line 150: why were there two more trials for each target condition during mechanical perturbation? This is a byproduct of our experimental design. We wanted to keep the number of trials within each block around 60 to avoid fatigue and wanted to have a proportion of mechanical and visual perturbation not too high to not bias participants’ behavior, the percentage of trials involving a mechanical perturbation and a visual change in target are respectively reported on lines 130 and 135 of the revised manuscript.

5) Line 194: Could you provide more details for how the statistical model was created? How were individual trials included in the model? Did you use the mean for each condition in the model? Please provide details so other researchers can replicate your analyses. We used all the trials to determine the effect of the target condition on the different parameters we explored and therefore never considered the mean value. Following the Reviewer’s suggestion, we added this information to the revised version of the manuscript (lines 198‐221).

Results

3) Figure 2: Could you add legends to panels 2a‐c? This has been added to the revised version of the figure

4) Line 227: for p values, you would typically report either p<0.05, p<0.01 or p<0.001 or the exact p‐ value. I would suggest reporting the exact p‐value.

We applied the method proposed by Benjamin and Berger to report p‐values (see lines 247‐248).

5) Line 228: “consistent with our” This has been changed

6) Line 284: same comment for p values as my previous comment See reply in point 4)

7) Lines 281‐287: I think these tests and the many following tests could be better represented in a tabular format.

Following this and the other reviewer’s comments, we changed the way we reported the statistical tests throughout the revised version of the manuscript. However, we believe that a tabular format does not necessarily improve the readability, and we hope that the updated report of the statistics allows a better navigation.

8) Line 288: I just noticed the abbreviation PD for posterior deltoid. Could you please use Post Delt or something else? I think PD should be reserved for Parkinson’s Disease. The abbreviations for posterior deltoid and pectoralis major have been respectively set to Post. Delt. and Pect. Maj. throughout the manuscript. 9) Line 292‐295: same tabular format would perhaps be better See reply to point 7)

10) Line 309: shallower modulation? Could you refer to the figure and the panel so it is easy to follow the authors’ thoughts. This has been clarified in the revised version of the manuscript (see lines 350‐351).

11) Line 312: Which “one” of the mechanical perturbations? I thought there is only one mechanical perturbation in each trial.

There was indeed a single mechanical perturbation per trial, this one was triggered at 6 and 8cm from the home target in experiments 1 and 2 respectively. The sentence has been rephrased to avoid this confusion (line 354).

12) Line 321: “reduce” This has been corrected.

13) Line 323: please label the figures. It is difficult to keep track of the figures without the numbers displayed on the figures. The revised version of the manuscript contains the figures in the text to make it easier to keep track of them.

14) Line 331 and 335: Now you are referring to muscles sometimes as PM and sometimes as Pectoralis Major. I think abbreviations like Pect Maj and Post. Delt are better because it does not force the reader to think what is being referred to here. The abbreviations for posterior deltoid and pectoralis major have been respectively set to Post. Delt. and Pect. Maj. throughout the manuscript.

15) Line 342: Do you mean 4G?

Thanks for pointing that out, this has been corrected.

16) Line 384: Why did you choose only the last 12? We wanted to verify whether the changes in behavior related to practice interfere with the modulation of EMG response with target condition. We selected the last 12 trials because it was the minimum amount of trials that allowed us to draw significant conclusion. Moreover, splitting the trials in half (12/24 for the slow and fast conditions) categorized them as early and late trials.

Discussion

1) Line 386: “task parameters” this is vague. Please elaborate briefly and clearly what was manipulated (target width) and the intent behind those manipulations? This has been clarified (line 427).

2) Line 401: Again, you have a specific experiment. You are modulating the width of the target at different rates. Using a generic phrase like “task parameters” just confuses the reader. Please be more specific.

We changed the term “task parameters” to “target width” to be more specific on line 446.

3) Line 409: Could you please put the inner loop in a different colour? The inner loop has been colored in gray, this has been indicated in the revised version of the manuscript.

Reviewer 2

The authors explore a task where participants make movements to a target that starts wide but can then switch to narrow or else shrink to narrow at one of two different speeds. Because their movement is occasionally perturbed to the right or left, participants must make an ongoing correction to hit the target and this correction increases in conditions where the target is narrower when it is hit. That is, there is least correction when the target stays wide, more for a slowly changing target, more even for a quickly changing target, and the most for a target that switches to a narrow target. The authors interpret this as an online adjustment of the control policy driven dynamically by changes in the conditions. This work follows up a similar study which only included the no change and switch conditions. The claim of the authors is that the fact that responses change also between the two conditions the target shrinks slowly or quickly shows that the participants are not merely switching between conditions but instead dynamically adjusting control parameters. I have a number of concerns about this work. They are organized in the order in which I view their importance from most to least. 1. I do not see why adjustments of the control policy are necessary. It seems to me that if target endpoints are represented in the state vector, then a single control policy would be sufficient. When target width changes, feedback response would automatically increase because the control policy would see a larger gap between current predicted position and target. Our developments are based on the minimum intervention principle (Todorov and Jordan 2002), which suggests that participants only correct deviations that interfere with task success during reaching movements. The question arises as whether the response modulation resulted from the MIP and the fact that participants exploited the structure of the goal, or from a single controller with different end‐point goals. We believe the latter hypothesis is not consistent with the two following experimental observations. First, when reaching to a wide target the hand path deviated to a new location with wider variance after mechanical perturbation which indicates the absence of eccentric aiming point in absence of perturbation (De Comite et al. 2021; Lowrey et al. 2017; Nashed et al. 2012). This is supported by the existence of a controller that counters the load to stabilize but not to correct endpoint hand coordinate. Second, even in the absence of perturbation the end‐point coordinates exhibited wider distribution which suggested that participants did not use a consistent aiming location (De Comite et al. 2021). These observations suggest that participants modulated the way they responded to deviations from the center of the target rather than aiming to a new specific endpoint. This point was clarified in the discussion section of the revised version of the manuscript on lines 434‐448.

2. Even if control policy is adjusted in the different conditions, I do not see how we can tell that the participants are adjusting it dynamically. They may be identifying which of the 4 conditions they are currently facing (based on assessment of the rate of change) and choosing one of the 4 prepared policies. The authors make this point themselves in lines 438‐440 and I did not understand how the lines following addressed this issue. They seem to move over to the question of intermediate strategies rather than addressing the possibility of a rapid switch in control.

This is a very important remark and we realize that this point must be discussed in more details. The hypothesis whereby participants identified which of the 4 conditions they were facing and switched to the appropriate control strategy cannot be formally rejected by the present study. However, since the feedback responses depended on the rate of change in target width and were independent of the target width at perturbation onset (lines 368‐390), it can be deduced that these adjustments occurred based on monitoring of dynamical variables, namely the rate of change of target width. In addition, the hypothesis of dynamical adjustment in control was also supported by the fact that we observed condition‐specific adjustments in behavior during the first trials of the fast and slow target conditions that participants did not encounter during training (lines 149‐150). Thus, they had not acquired a controller tuned to this specific perturbation at that stage. This discussion was also added to the revised version of the manuscript (see lines 493‐507).

3. It was not clear to me what the analysis of EMG adds to the paper. Ultimately, if movements change then EMG must as well. The question that the EMG is supposed to answer could be made clearer. From the discussion and the statistical approach, I came to the conclusion that the changes in EMG where meant to test for the difference between adjustments based on changes in the control strategy (during the LL period of 50‐100 ms) and voluntary changes in intent (during the VOL period of 100‐180 ms). I’m not at all certain that this interpretation is correct. If so, it might represent a response to my point 1. On the other hand, the changes in the EMG in the LL period are quite small compared to the changes during the VOL period and do not seem to be consistent across conditions. This would suggest that change in movement path is driven largely (if perhaps not entirely) by the EMG changes in the VOL period. Almost all of their behavioral effect is then explained by the VOL period EMG. Again, I am not sure I understand well enough to know how this affects the overall interpretation. Perhaps the authors could make this clearer. The reason why we investigated the EMG responses was that we wanted to determine the onset of changes in EMG as the timing of a motor response sets constraints on the underlying neural pathways. The data reported in the present study demonstrates that both long‐latency (LL) and early voluntary responses contributed to the change in behavior that we documented. Even if the modulation of the LL responses on its own was small, the fact that they were modulated means that the onset of response modulation was visible in this epoch, which is compatible with the idea that this feedback control system implements goal‐directed state‐feedback control (Crevecoeur and Kurtzer 2018). This point has been clarified in the revised version of the manuscript (lines 298‐301).

4. I have concerns about the model that was used for assessing significant differences. Throughout the text, the authors report beta1 as a single number, suggesting that the effect of condition is modeled as a regressor rather than as 4 separate conditions. If that is right, this would be a mistake. Since the authors are using fitlme, the key point is that condition in the matlab table needs to be specified as a categorical variable. I may be wrong about what they’ve done, but otherwise I can’t make sense of having a single parameter give the effect size for condition. This is an important point that required clarification. We indeed used a continuous predictor for the target condition because we wanted to have a proxy of the change in target width (smaller values for this predictor corresponded to narrower final targets). This variable can be seen as a non‐linear transform of the level of constraints and the value of βଵ as a slope is interpretable in the sense that a narrower target is associated with a stronger response. We understand the reviewer concern and rerun all our models using a categorical predictor for the target condition. This updated model provided conclusions similar to that with continuous predictor for each analysis performed in the manuscript. We added this justification to the manuscript and reported that all results performed with a categorical predictor supported the same conclusions. This is mentioned in the revised version of the manuscript on lines 198‐221.

5. It is also not clear that from the text that they have properly specified the repeated measures character of the experimental design. That is, the formula between lines 194 and 195 does not seem to explicitly specify that this is a repeated measure design and traditionally this is not how it would be written. This might explain the error bars in Figure 4C‐F that seem to me to be suspiciously small. We completed the equation (line 202) to properly specify the repeated measures of the experimental inter‐individual differences (captured by the term α) and the randomness within a single participant (captured by ϵ). In this model, the fact that measurements are repeated for each individual is captured by the random term of the mixed model.

6. The follow up paired tests between groups seem to be done using a direct paired test instead of a comparison between parameters of the regression. If so, this would also be a mistaken approach. Post hoc comparisons following a regression should be performed on the parameters of the regression (that is looking at contrasts of the dummy variables representing the different groups in the categorical variable for condition). Compounding this, the authors seem to be using a nonparametric (ranksum) paired comparison when the regression was specified as a parametric analysis. This choice is not justified and seems arbitrary. We understand the Reviewer’s concern and modified the paper accordingly. The posthoc tests performed in the revised version of the manuscript are similar mixed models than those used to characterized the main effect except that only two target conditions (instead of four) were used as a predictor. This was clarified in the Methods section (lines 218‐221) and the reported posthoc tests are corrected throughout the Results section of the revised version of the manuscript.

7. The authors should provide effect size and confidence intervals for comparisons between conditions. The revised version of the manuscript contains the standard deviation for each fitted parameter and the effect sizes (computed as the Cohen’s d) associated with each comparison.

8. In the analysis of the EMG, the authors interpret the difference between a significant and a non‐ significant result to mean that there is a meaningful difference between the outcomes (such as on line 281 but in other place as well). They also sometimes treat a non‐significant result as an indication of lack of effect (such as on line 333). It is important only to draw conclusions following a direct statistical test of the claim being made and not to draw conclusions from lack of significance (unless an explicit test for equivalence or some other clearly stated criterion for equivalence is used). We thank the reviewer for making this remark. We make sure that in the revised version of the manuscript we did not interpret non‐significant results as a lack of effect.

9. When providing errors in the text, the authors do not make clear if they are standard errors of the parameter or its confidence interval. The error reported in the text are standard errors of the parameters, this has been clarified in the Methods section of manuscript (line 209).

10. The authors should add errobar patches like those in Figure 2 wherever it makes sense. This includes Figure 3A‐D and Figure 4G. We understand this concern but the within and between subjects underlying variability of EMG signals is difficult to represent graphically. Indeed, there are two levels of variability: one capture by the individual residuals and the other by the random offset associated with each participant. If we compute and represent the standard deviation of this EMG activity, the variability presented on the figure will not correspond to that captured by the statistical models and could mislead the reader’s interpretations. For this reason, we decided to only represent the variability in the EMG signals as small insets located in each figure representing EMG traces (see Figures 3 and 4).

11. In Figure 3E‐H, it is very hard to resolve the data from the different subjects. I recommend using slightly different grayscale or color to allow subjects to be better differentiated and also smaller dots for the markers. In addition, you can jitter the x position of the different subjects slightly to help make them more visible. This is a very good point that has been implemented in the revised version of Figure 3.

12. The figure legends are formatted centered. I think that might be an oversight. Personally, I would find it much easier to review the figures if the figure legends appeared following the figures instead of needing to jump back and forth.

In the revised version of the manuscript, we put the figures in the text with their associated legends
